# Athlete Preferences for Nutrition Education: Development of and Findings from a Quantitative Survey

**DOI:** 10.3390/nu15112519

**Published:** 2023-05-29

**Authors:** Hayley Solly, Claire E. Badenhorst, Matson McCauley, Gary J. Slater, Janelle A. Gifford, Bevan Erueti, Kathryn L. Beck

**Affiliations:** 1 School of Sport Exercise and Nutrition, College of Health, Massey University, Private Bag 102904, Auckland 0745, New Zealand; hayleymsolly@gmail.com (H.S.); c.badenhorst@massey.ac.nz (C.E.B.); matson.mcc@gmail.com (M.M.); 2 School of Health, University of the Sunshine Coast, Sippy Downs, QLD 4556, Australia; gslater@usc.edu.au; 3 Australia and Australian Institute of Sport, Bruce, ACT 2617, Australia; 4 Discipline of Exercise and Sport Science, Faculty of Medicine and Health, The University of Sydney, Sydney, NSW 2006, Australia; janelle.gifford@sydney.edu.au; 5 School of Health Sciences, College of Health, Massey University, Palmerston North 4472, New Zealand; b.erueti@massey.ac.nz

**Keywords:** diet, education, sport, elite, survey, behaviour change

## Abstract

Nutrition education (NE) is one of several strategies aimed at enhancing the dietary intake of athletes. This study investigated NE preferences of New Zealand and Australian athletes competing nationally and internationally. Athletes (*n* = 124, 22 (18, 27) years, female 54.8%) from 22 sports completed an online survey, with responses analysed using descriptive statistics. Teaching techniques considered ‘extremely effective’ were life examples (47.6% of athletes), hands-on activities (30.6%), and discussions with a facilitator (30.6%). Setting personal nutrition goals was important to most athletes (83.9%), along with two-way feedback with a facilitator (75.0%). General nutrition topics considered ‘essential’ were energy requirements (52.9%), hydration (52.9%), and nutrient deficiencies (43.3%). Performance topics considered ‘essential’ were recovery (58.1%), pre-exercise nutrition (51.6%), nutrition during exercise (50.0%), and energy requirements for training (49.2%). Athletes preferred a ‘combination of in-person group and one-on-one sessions’ (25% of athletes), ‘one-on one sessions’ (19.2%) and ‘in-person group sessions’ (18.3%), with only 13.3% interested in ‘exclusively online delivery’. Sessions of 31–60 min (61.3% of athletes) held monthly (37.5%) and undertaken with athletes of the same sporting calibre (61.3%) were favoured by the participants. The preferred facilitator was a performance dietitian or nutritionist (82.1% of athletes), who had knowledge of the sport (85.5%), experience in sports nutrition (76.6%), and credibility (73.4%). This research provides novel insights into the factors that need to be considered when designing and implementing nutrition education for athletes.

## 1. Introduction

High-quality dietary intake is important for an athlete’s performance and overall health status [1]. Sports nutrition guidelines recommend athletes consume a healthy diet in combination with specific sports nutrition strategies relevant to training and competition schedules [1,2]. However, evidence suggests that athletes often lack adequate fruit and vegetable intake and consume higher-than-recommended intakes of discretionary foods [3,4,5]. Several factors influence an athlete’s ability to make quality food choice decisions, including convenience, finances, taste preferences, body image, culture, lifestyle, health beliefs, weight control, and gastrointestinal discomfort [6,7,8]. A factor that may not only influence the dietary intake of athletes but may also influence several of the factors related to food choice is the athlete’s nutrition knowledge [2,7].

Previous research has pointed to gaps in the nutrition knowledge of athletes [9,10], which if resolved may facilitate an improvement in quality food choices. Therefore, a key role of performance nutritionists working with athletes is to facilitate improvements in nutritional knowledge through the provision of nutrition education (NE) [1]. In a systematic review, Tam et al. [10] found over 80% of NE interventions improved nutrition knowledge in athletes. While NE interventions do not always ensure a change in dietary practice [11], evidence suggests athletes may benefit from NE through increases in nutrition knowledge, improved eating habits, and changes in body composition [12]. Incorporating other factors that may influence the behaviour of athletes (e.g., support from others, availability of healthy food) could help to optimize NE [13].

Currently, there are a range of methods used to deliver NE interventions, which impacts nutritionists’ and sporting organization’s decisions on what design elements and content to include for the most effective NE [10]. In addition, more recent research would suggest that there may be the need for generation-specific pedagogy to support differences in knowledge construction for athletes [14]. For example, ‘Millennials’ prefer visual leaning tools (e.g., videos), and expect content to be ‘fresh and current’, while ‘Baby Boomers’ have stronger attention spans and appreciate repetition [15]. Furthermore, with increased access to online information, NE should be adapted to new methods and preferences of communication to ensure it is well received by athletes [16,17].

Whilst preferences for NE have been explored qualitatively and quantitatively in non-athlete groups [18,19], investigations regarding athlete preferences for NE have only recently been explored [20,21]. In qualitative interviews of 12 professional Australian football players, athletes preferred one-on-one consultations with a sports dietitian when asked about the types of NE that would help them (i.e., one-on-one consultation, group sessions, cooking classes) [21]. A survey of young Canadian athletes found individual consultations (69%), presentations (63%), and the internet (40%) were the preferred means of NE [20]. These studies are limited as NE preferences of athletes were not the focus of the investigation, with only generic questions regarding athlete NE preferences provided. Recent qualitative research in 20 highly trained/elite New Zealand athletes found these athletes preferred NE of six months duration, undertaken in small groups, but no quantitative data was obtained [22]. To date, limited research has been undertaken in high-performing athletes to obtain a broad yet comprehensive understanding of athlete NE preferences including pedagogy, content, format, and facilitator. Further research is needed to quantify athlete preferences in NE to enable nutritionists, dietitians, and sporting organizations to implement effective NE programmes. This research therefore aimed to develop and implement a survey to obtain a comprehensive understanding of athlete preferences for NE.

## 2. Methods

### 2.1. Study Design and Participants

The study is a descriptive cross-sectional analysis where participants completed a survey developed by the research team exploring athlete preferences for NE. Recruited participants were over 16 years of age (parental consent to participate is not required at this age) and tier 3 (elite, competing at international level) and tier 4 (highly trained, competing at national level) based on the classification framework by McKay et al. [23]. Ethics approval was granted by the Massey University Human Ethics Committee (Southern B, Application SOB 20/02 and Northern, Application NOR 21/38) with Reciprocal Approval from the Australian Institute of Sport (AIS) Ethics Committee. Prior to completing the survey, participants completed an online consent form and a brief screening questionnaire to ensure eligibility.

Recruitment was undertaken using convenience sampling. In New Zealand, the survey was shared through existing industry contacts and advertising on social media channels (Facebook, Instagram, and LinkedIn). In Australia, sports nutrition practitioners across the National Institute Network (NIN) were informed of the research and interested practitioners forwarded the survey to athletes within their network. Data were collected from early September through to late November in 2021.

### 2.2. Development of Athlete Nutrition Education Preferences Survey

A draft survey was developed by the lead researcher (HJ, an ex-athlete and nutrition and dietetic student). Apart from the questions on sports/demographics, all survey questions were developed from scratch. The development of survey topics and questions was heavily informed by the literature, including the use of resources for the development of online surveys [24,25], questionnaire topics used in related research [16,18,19,26,27], and results from earlier investigations of athlete preferences for education in both nutrition [20,21] and other topics (e.g., concussion) [28,29]. Findings and questions from focus groups exploring New Zealand athlete preferences for NE were also used to develop the survey [22]. Specific areas of interest were pedagogy, content, format, and facilitator, which were based on a conceptual model developed for promoting physical activity intervention design and delivery, which also applies to other health behaviours [30]. The researchers who developed this model suggest that if the core intervention components (pedagogy, content, format, and facilitator) of the conceptual model are aligned with participant preferences and attributes, participants will experience greater engagement and improved outcomes. With tailored design and delivery of interventions and education, this will ultimately lead to improved acceptability and efficacy [30]. The survey was sent to the research group (comprising three experienced sports dietitians (GS, JG, KB), a sport and exercise physiologist (CB), and a nutrition and dietetics graduate (MM) to ensure content validity. Recommendations range from two to twenty content experts to review newly developed questionnaires, with at least five experts recommended to ensure sufficient control over agreement occurring by chance [31]. The survey went through over 25 iterations with content experts making edits and suggestions to the questionnaire via email until there were no further comments or suggestions. Where there was difficulty reaching consensus regarding the content or format of the survey, verbal discussion occurred between the content experts in order to reach agreement.

Pre-testing was undertaken with a convenience sample of 10 experienced ex-athletes (tier 3 and tier 4) [23] to ensure the survey’s relevance to athletes, as well as three Massey University nutrition staff/students to further establish content validity and ensure readability. Feedback from both groups was received either verbally or by email. Where necessary, these participants were prompted to expand and explain their responses until no new input was obtained.

Prior to pilot testing, the survey took approximately 20–25 min to complete. Following adaptation (mainly including more lay terminology; avoidance of repetition; ensuring all questions were directly relevant to the research objectives; and use of unipolar rather than bipolar Likert scales), 20 New Zealand-based tier 3 and tier 4 athletes [23] were invited to pilot-test the survey. The completed responses (*n* = 11; 7 females) were athletes from badminton (*n* = 4), athletics (*n* = 2), motorsport (*n* = 2), water polo, swimming, and soccer (all *n* = 1), who all provided feedback via email. Changes made following this feedback included rephrasing of questions for participant comprehension and shortening the survey.

Changes to reduce the survey length included consolidation of questions/topics in the pedagogy section to ensure all questions/topics were relevant and different; reducing the thought processes for some questions by asking athletes to “check all that apply” rather than asking participants to rank in order; and asking participants to rank only the top three statements in ranked order questions. Athletes involved in pre- and pilot testing did not participate in the final survey.

The final survey consisted of six parts. Part 1 included questions on level of interest in performance nutrition (1 question: 5-point Likert scale ranging from ‘not interested at all’ to ‘extremely interested’) and previous sources of NE (1 multiple choice question). Parts 2–6 covered preferences for pedagogy: content; format; facilitator; and a sport/demographic section. The section on pedagogy aimed to understand participant preferences for effective teaching techniques (19 techniques: 5-point Likert scales ranging from ‘not at all effective’ to ‘extremely effective’), along with the importance of the use of various feedback and monitoring strategies (3 items: 5-point Likert scales ranging from ‘not at all important’ to ‘extremely important’), as well as two questions regarding whether enjoyment and engagement were needed for success in NE (5-point Likert scales ranging from ‘never’ to ‘always’). For content, participants were asked how much they prioritised curricular topics related to general (12 topics: 5-point Likert scales ranging from ‘not a priority’ to ‘essential’), performance (9 topics: 5-point Likert scales), and skills related nutrition information (11 topics: 5-point Likert scales), as outlined in the model by Parks et al. [32]. Two questions regarding preferences for content characteristics (credibility and use of repetition) for the success of NE were also included (5-point Likert scales ranging from ‘never’ to ‘always’). The format section asked about preferences for general delivery (three multiple choice questions and one top-three ranking question). For both in-person and online delivery sessions, there were multiple choice questions regarding session duration, frequency, ideal number of participants, and the total number of sessions preferred (total of eight questions). For online delivery sessions, participants were asked to rank their top three online delivery methods (from eight options), with a further ranking question regarding preferences for live or recorded sessions. The facilitator section comprised multiple choice questions aimed at quantifying the athlete’s preferences for personal attributes (10 attributes), relatability (8 characteristics), and qualifications and experience (8 options) in a facilitator. Participants were asked to rank their top three preferences for a facilitator (from 6 options), as well as whether they wanted the same person to deliver nutrition education sessions (5-point Likert scale ranging from ‘never, different facilitators are good’ to ‘all of the time’). For some questions, athletes were able to select an ‘unsure’ response to help avoid guessing. The survey was delivered online (Qualtrics 2021, version June 2021), and pilot data showed that the final survey took approximately 15–20 min to complete.

### 2.3. Statistical Analysis

Data was entered and analysed using IBM SPSS Statistics (version 27.0). Descriptive statistics (i.e., frequency counts, percentages for categorical data and median (25th, 75th percentile) for non-parametric data) were calculated for all variables following normality testing of the data.

## 3. Results

### 3.1. Participant Characteristics

Of 138 completed responses, two were <16 years of age, 11 were not competing at a tier 3 or 4 level, and one did not provide consent, leaving 124 responses for analysis. Mean completion time of the survey was 18.4 ± 8.4 min. Of the 124 athletes, 54.8% were female; 101 athletes lived in New Zealand (81.5%), 19 in Australia (15.3%), and four elsewhere (3.2%). Over half (55.6%) had completed or were completing a university qualification, with 8.9% undertaking nutrition related university studies. Athletes mainly competed in rowing (26.6%), athletics (8.9%), and cycling (8.9%) (Table 1). Athletes (83.1%) were ‘very or extremely interested’ in performance nutrition. Common sources of nutrition information were coaches (71.8%), other athletes (69.4%), specialist sports dietitians or performance nutritionists (65.3%), and social media platforms (63.7%).

### 3.2. Pedagogy

Most athletes thought NE should be engaging (96%) and enjoyable (81.4%) ‘always’ or ‘often’. Athletes (83.9%) thought setting goals for their nutrition within NE sessions was ‘very’ or ‘extremely’ important. The need for these goals to be monitored by the facilitator was rated as ‘very’ or ‘extremely’ important by 53.2% of athletes, while 75.0% ranked receiving some two-way feedback between themselves and the facilitator as ‘very’ or ‘extremely’ important.

**Figure 1:**
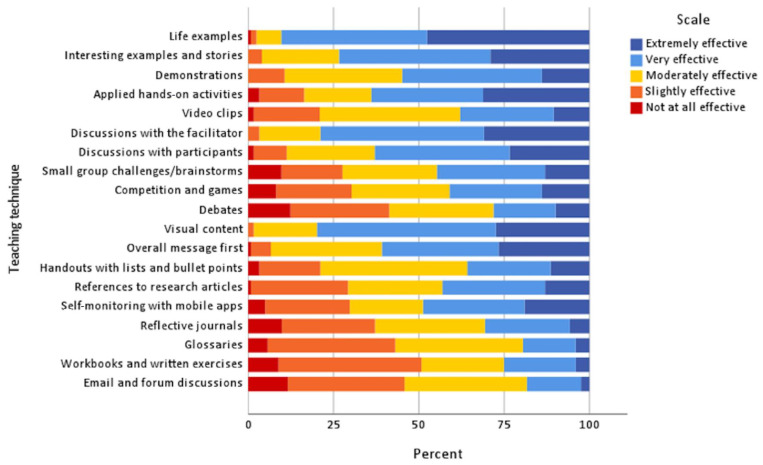
The percentage of athletes (*n* = 124), who rated their perceived effectiveness of teaching techniques for learning in NE. Techniques are grouped by learning styles: kinaesthetic, aural, visual, reading, and writing. Note: not shown are unsure responses (*n* = 23, 1%) for this section.

Preferred teaching techniques were a mixture of kinaesthetic (e.g., demonstrations, applied ‘hands-on’ activities), aural (e.g., open discussions with the facilitator), and visual techniques (e.g., charts, diagrams, infographics). Teaching techniques related to reading, writing, or recording (e.g., self-monitoring with mobile applications, workbooks) were less preferred. Teaching techniques involving competition (e.g., competitions/games, debates) showed a mixed response (Figure 1).

### 3.3. Content

**Figure 2:**
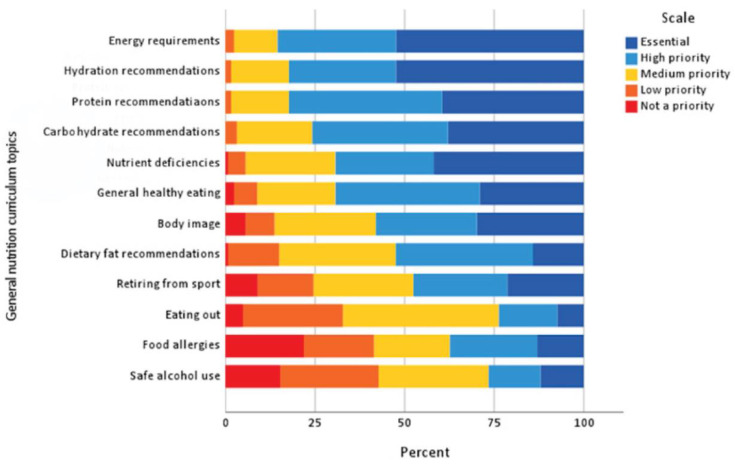
Priority of general nutrition curriculum topics to be included in NE (*n* = 124). Note: not shown are unsure responses (*n* = 9, 0.6%) for this section.

**Figure 3:**
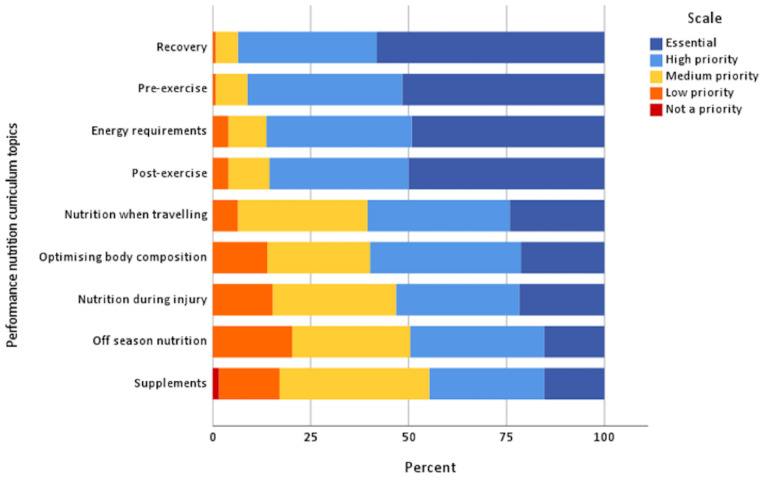
Priority of performance nutrition curriculum topics to be included in NE (*n* = 124). Note: not shown are unsure responses (*n* = 4, 0.4%) for this section.

**Figure 4:**
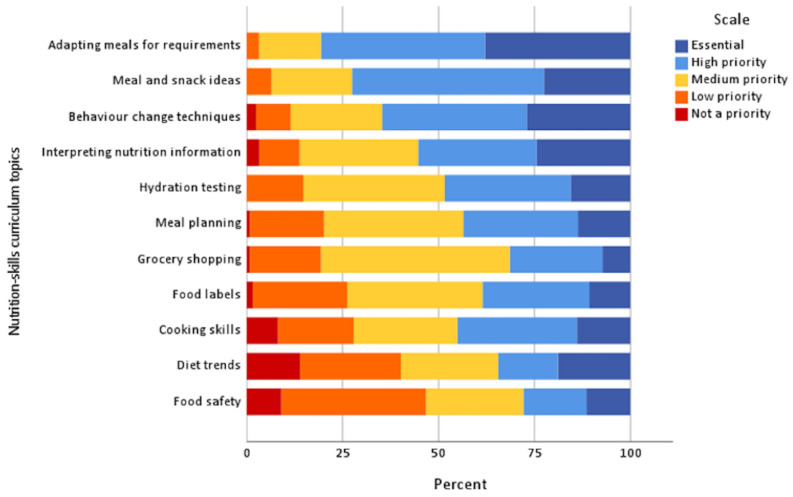
Priority of nutrition-related life skills to be included in NE (*n* = 104). Note: not shown are unsure responses (*n* = 11, 0.8%) for this section.

Credible content in NE was important for 91.1% of athletes ‘always’ or ‘often’. Most (69.4%) athletes felt topics should be repeated ‘sometimes’. General nutrition topics considered ‘essential’ were energy requirements (52.9% of athletes), hydration (52.9%) and nutrient deficiencies (43.3%). ‘Essential’ performance nutrition topics were recovery nutrition (58.1%), before and during exercise nutrition (51.6 and 50%, respectively), as well as overall energy requirements (49.2%). Adapting meals for training requirements (37.9%) and behaviour change techniques (26.6%) were considered ‘essential’ nutrition-related life skills (see Figure 2, Figure 3 and Figure 4).

### 3.4. Format

Athletes preferred a ‘combination of in-person group and one-on-one sessions’ (25% of athletes), ‘one-on one sessions’ (19.2%), and ‘in-person group sessions’ (18.3%). The least preferred delivery settings were ‘exclusively online delivery’ (13.3%), ‘one-on-one sessions combined with online delivery’ (7.5%), and ‘in-person group sessions combined with online delivery’ (2.5%). Webinars (65.8% of athletes) ranked as the preferred online delivery method. Less than ten percent of athletes ranked emails (7.3%), podcasts (6.5%), social media (5.7%), websites (5.7%), apps (4.1%), and forums (2.4%) as their preferred online delivery method. Athletes preferred that NE group sessions be undertaken with athletes of the same sporting calibre (61.3% of athletes), of similar age (52.4%), with teammates, and with coaches (both 51.6%) (Table 2).

Approximately half of respondents indicated a preference for NE to run continuously throughout the year (50.8%), rather than intermittently (29.0%) or as a short course (16.1%). For those athletes who did not prefer the NE to be continuous (45.2%), their preferences were for it to occur at the beginning of season (21.8%) or the off season (16.1%). Most athletes (62.1%) preferred smaller numbers in group sessions (up to 10 people). The most preferred duration and frequency for a single session was 31–60 min (61.3% of athletes), held once per month (37.5%) or once every two (29.0%) months. Overall, 1–10 sessions were favoured (74.6%) (Table 3).

### 3.5. Facilitator

Table 4 details the preferred qualities of the NE facilitator. Briefly, the preferred facilitator was a sports dietitian or nutritionist (82.1% of athletes), and most athletes (82.3%) wanted the same facilitator ‘all’ or ‘most’ of the time. Athletes preferred the facilitator be credible (73.4%), relatable and likeable (both 66.9%), knowledgeable in the sport in which they were providing education (85.5%), and willing to learn more about the sport (71.0%). Finally, a credible facilitator had experience in sports nutrition (76.6%) and/or was a registered nutrition professional (67.7%) (see Table 4).

## 4. Discussion

### 4.1. Overall Findings

To our knowledge, this is the first study to quantify elite and highly trained athletes’ preferences in NE across the areas of pedagogy, content, format, and facilitator. Athletes preferred engaging NE taught through a mixture of kinaesthetic, aural, and visual techniques. Athletes prioritised key topics (energy, nutrient, and hydration requirements; pre-training, post-training, and recovery nutrition) pertaining to general and performance nutrition, with adapting meal requirements being the most valued nutrition-related life skill. A combination of group and one-on-one sessions was favoured. Preferred length of sessions were 31 to 60 min, occurring every one to two months as part of a continuously running NE programme. Finally, athletes valued a credible facilitator such as a performance dietitian or nutritionist with professional experience who was knowledgeable in the sport and non-judgmental.

### 4.2. Pedagogy Preferences

Perception of what is considered an engaging and effective teaching technique will likely depend on the preferred learning style of the individual. The most preferred teaching techniques in this study were aligned with a kinaesthetic learning profile (e.g., life examples, the use of interesting examples or stories) and learning through discussion and engagement. Braakhuis et al. [27] also found a kinaesthetic learning style was the most dominant in 93 athletes who completed the visual, aural, read/write, kinaesthetic (VARK) questionnaire. Using focus groups, McCauley [22] found athletes preferred kinaesthetic teaching techniques such as stories and real-life examples. Similar to the results of McCauley [22], most athletes in this study did not favour reading or written tasks such as the use of workbooks and written exercises. Likewise, previous research in sports nutritionists noted that 77% used ‘pictures and infographics’ and stated that these were the preferred type of content delivered through their social media [17]. Interestingly, information presented visually, such as ‘charts, diagrams, and infographics’, was also preferred by athletes in this study. Current and previous research on visual content to support NE by sports nutrition practitioners may be a relatively easy teaching method to adopt.

Setting performance nutrition goals within the NE session was perceived to be important for almost all athletes in the present study. Involving athletes in planning has been shown to facilitate athlete motivation and intervention compliance [33]. For nearly all athletes, it was important that NE be both engaging and enjoyable, and most wanted two-way feedback with the NE facilitator. Taking time to understand the athletes’ preferred learning styles can be invaluable in designing what teaching strategies to utilise within NE. This could be facilitated informally or through questionnaires such as the VARK questionnaire for athletes [26]. It may be that a range of teaching mediums is needed to encompass different learning styles within a group or team of athletes.

### 4.3. Content Preferences

The inclusion of activities such as cooking classes, supermarket tours, and nutrition-related calculations are known to improve athlete knowledge, capabilities, and dietary behaviour [34,35,36,37]. Teaching these skills through practical opportunities improves self-efficacy, or a belief in the athlete’s own capacity to perform a skill, thus improving behaviour change outcomes [38,39]. Prioritised skills-based topics within the current study were ‘adapting meals to suit performance requirements’ and ‘meal and snack ideas’, while fewer athletes considered ‘cooking skills’ and ‘grocery shopping’ to be a priority. This conflicts somewhat with the athletes in this study preferring teaching techniques using a kinaesthetic approach but may be because most of the athletes took self- or shared responsibility for cooking and purchasing food, and they may have felt they already had skills in these areas. Consequently, there was a stronger preference for education on how to select and compose the most appropriate meals and snacks to support performance needs.

Athletes wanted information on energy, macro- and micronutrient requirements, and hydration, with a preference for performance nutrition topics focusing on before and during exercise and recovery nutrition. These topics are fundamental components of basic sports nutrition recommendations [1], which are important for athletes to understand before focusing on more specialised sports nutrition topics (e.g., supplement use, optimising body composition). Within this study, there were mixed responses regarding ‘navigating diet trends’ which may have been covered in the survey statement ‘interpreting nutrition misinformation’. Sports NE often focuses on the manipulation of body composition, yet preferences regarding the topic ‘optimising body composition’ were not considered essential by most athletes.

Other studies have shown athletes prefer a diverse education curriculum which includes discussions on interpreting nutrition information [22] and behavioural change techniques [38]. Expanding NE to include other factors influencing dietary behaviour, such as by using tools such as the Athlete Food Choice Questionnaire (AFCQ) [40], can aid practitioners in more tailored and impactful NE for their athletes. Online tools such as the Accelerated Sports Nutrition Assessment Platform (ASNAP) afford an exploration of current knowledge as well as practice (as inferred via diet quality) to effectively target NE content [41,42]. Such tools could be used to identify current gaps in knowledge and dietary intake. Re-administration of such tools pre- and post-NE may provide an opportunity to ascertain the efficacy of the intervention, which may be important when rationalising investment in nutrition care.

### 4.4. Format and Facilitator Preferences

Athletes preferred in-person group and one-on-one sessions combined or separately. Qualitative studies found professional Australian footballers who had free access to a sports dietitian preferred one-on-one consultations with a sports dietitian [21], while highly trained/elite athletes from a range of sports in New Zealand preferred in-person group sessions [22]. It was not determined whether the athletes in the study by McCauley [22] had previous access to a sports nutritionist or dietitian. A large study of young Canadian athletes ranked individual consultations, presentations, and the internet as their preferred sources for obtaining information on supplements, while their primary sources of NE were family and friends, coaches, and athletic trainers [20]. Within the current study, two-thirds of athletes had received NE from a sports dietitian or nutritionist; however, the format in which this support and education was received was not clear. This may explain the spread of preferences across both group and one-on-one sessions, which could be influenced by previous NE. Most NE interventions in the literature focus on either individual or group session formats of a heterogeneous nature, making them difficult to quantify and compare [10,11]. For a mixed-methods approach to NE, there is less evidence on beneficial parameters. Despite a tailored curriculum, one-on-one sessions partnered with group lectures did not consistently improve dietary habits in adult athletes [43]. More recently, a 16-week intervention of weekly online lectures and nutrition counselling every second week improved sports nutrition knowledge and suggested weak evidence for improved sports nutrition behaviour in female endurance athletes with symptoms of Relative Energy Deficiency Syndrome [44]. Athletes in the current study ranked their preference for solely online delivery of NE as low. Research in tertiary students found that while online learning appeared to be more effective for short-term knowledge acquisition, a mixture of in-person and interactive online delivery methods was significantly more effective than asynchronous delivery methods for knowledge retention [45]. This aligns with social presence theory which proposes that in-person interaction is crucial for quality learning [46]. Despite athletes commonly using social media for nutrition information [16], findings from this survey found social media was not a preferred online delivery platform, and webinars were preferred. Indeed, athletes with access to performance nutrition practitioners in one study were less inclined to seek nutrition information via social media, and athletes had some concerns about the reliability of information available through social media [16].

Overall, athletes preferred up to 10 NE sessions held every one or two months. While preferred duration of NE was not specifically considered in the current study, a recent systematic review in non-athletes showed that one of the major factors for successful behavioural changes for good health was a NE programme duration greater than or equal to five months [47]. However, there are few NE interventions in athletes of equal or greater duration in the current literature [10,11]. In college athletes, an NE programme of five months’ duration improved overall nutrition knowledge but not dietary intake [48], while another two-month-long NE programme found significant improvements in dietary choices, self-efficacy, and nutrition knowledge [39]. Further research is needed to fully understand optimal delivery method and duration for NE to ensure it has a positive impact on both the knowledge and dietary practices of athletes.

The performance nutritionist is responsible for a breadth of activities [1] and for efficiency may offer group education sessions with individual attention triaged to athletes most in need. Most athletes wanted to undertake NE with athletes of the same sporting calibre. A preference to learn with teammates was not likely to apply to athletes in individual sports. Coaches were also some of the most preferred people to participate in NE alongside athletes, and similarly to other studies [49,50], coaches were found to be the main source of nutrition information for athletes. Nutrition knowledge in coaches has been found to be better than that of athletes [49,51]; however, the nutrition knowledge and training of coaches in facilitating positive athlete behaviour change regarding nutrition is insufficient [38,52,53,54]. It is advised that the coaches’ role in NE should therefore be regarded as reflecting athlete preferences of participation rather than facilitation. Athletes’ value of credible facilitators, such as sports dietitians, has been reported elsewhere [55]. Synonymous with the findings from the present study, recent research has found that athletes have a higher preference for a credible facilitator who has knowledge of the athlete’s sport, a preference that diverges from the previous rhetoric that the nutrition facilitator needed to look ‘athletic’ to gain the athlete’s trust [22,56]. The facilitator’s credibility as a professional with sports nutrition experience and knowledge of the sport should all be considered when designing athlete NE.

### 4.5. Implications of Findings

This research provides insights that dietitians and nutritionists can use to develop nutrition education interventions for athletes. More research is needed to investigate and compare other cohorts of athletes (e.g., recreational athletes, high school, college or Master’s athletes; athletes from different sports; athletes from different countries and cultures; and athletes with little interest in nutrition) as nutrition education preferences for these athletes may be different. The findings of this study set the stage for a randomised controlled trial to be conducted to investigate the efficacy of various nutrition education modalities and platforms for improving nutrition knowledge and improving dietary behaviour in athletes.

### 4.6. Study Strengths and Limitations

While the study was able to recruit a good sample size of athletes from a broad spectrum of sports, there were some limitations. Setbacks to distributing the survey included athlete and practitioner commitments in an Olympic year, as well as recruitment being undertaken during strict government lockdown restrictions within Auckland, New Zealand due to the COVID-19 outbreak. During this period, many athletes were unable to meet in person and had to use online methods of learning and communication. Previously online methods of communication may have been seen as novel; however, during this time it is a possibility that participants were experiencing screen fatigue [57] which may have influenced their NE delivery preferences. The survey was designed to be easy to complete by the athletes yet thorough enough in detail to accommodate substantial findings on the design of a quality NE. However, it is possible that the survey length may have been a barrier to obtaining greater response rates. Athlete recruitment was restricted to the researchers’ connections with certain sports and is therefore not a complete representation of all sports within Australia and New Zealand. With volunteer sampling, it is more likely that athletes with a higher interest in nutrition (83.1% ‘very’ or ‘extremely’ interested) were more inclined to participate; as such, the possibility of the result reflecting some sample bias needs to be considered. While the content validity of the survey was assessed, further research should be undertaken to assess the reliability of the survey by having the same group of athletes complete the survey on separate occasions to ensure the answers provided do not differ.

## 5. Conclusions

Athletes wanted engaging and enjoyable NE taught through a mixture of kinaesthetic, aural, and visual techniques. They thought setting goals was important and valued two-way feedback with the facilitator. Athletes wanted credible education on energy, macro- and micronutrient, and hydration needs, as well as pre-, during- and post- (recovery) exercise requirements. Including nutrition-related life-skills in NE, such as how to adapt meals to meet nutrition requirements, was a high priority for most athletes. Athletes preferred a combination of in-person group and one-on-one sessions. For group sessions, having small groups of up to 10 athletes of the same sporting calibre was preferred. For most athletes, NE sessions would be 31 to 60 min and held every one to two months. A performance dietitian or nutritionist with experience in sports nutrition was the preferred facilitator. Being knowledgeable, relatable, likeable, and non-judgmental as well as willing to learn more about the sport were also important characteristics of the facilitator. Further research should be undertaken to investigate whether there are differences in NE design and content preferences of athletes based on age, sport, calibre (e.g., high school athletes), and participation in previous NE. More research is needed to investigate NE preferences of athletes who live in different countries and cultures, as well as those who have less of an interest in performance nutrition.

## Figures and Tables

**Table 1:** Athlete Characteristics (*n* = 124).

Variable		Participants, *n* (%)
Age in years		
	Median (25th, 75th percentile)	22.0 (18, 27)
Gender		
	Female	68 (54.8)
	Male	50 (40.3)
	Not answered	6 (4.8)
Highest education level ^a^		
	School ≤ year 11	4 (3.2)
	School year 12 or 13	40 (32.3)
	Polytechnic or apprenticeship	4 (3.2)
	University	69 (55.6)
	Not answered	7 (5.6)
Current living situation		
	With family	48 (38.7)
	With partner/spouse	31 (25.0)
	With housemates	29 (23.4)
	Alone	6 (4.8)
	Boarding school/hostel	4 (3.2)
	Not answered	6 (4.8)
Food purchasing responsibility		
	Self	51 (41.1)
	Another household member	29 (23.4)
	Shared responsibility	34 (27.4)
	Food service organization ^b^	4 (3.2)
	Not answered	6 (4.8)
Food preparation/cooking responsibility		
	Self	47 (37.9)
	Another household member	14 (11.3)
	Shared responsibility	54 (43.5)
	Food service organization	3 (2.4)
	Not answered	6 (4.8)
Special dietary requirements ^c^		
	Yes	24 (19.4)
	No	94 (75.8)
	Not answered	6 (4.8)
Sport ^d^		
	Rowing	33 (26.6)
	Athletics	11 (8.9)
	Cycling	11 (8.9)
	Triathlon	9 (7.3)
	Kayaking/canoeing	8 (6.5)
	Skating	8 (6.5)
	Rugby	7 (5.6)
	Auto racing	6 (4.8)
	Swimming	6 (4.8)
	Sailing	4 (3.2)
	Hockey	3 (2.4)
	Table tennis	3 (2.4)
	Basketball	2 (1.6)
	Para-equestrian	2 (1.6)
	Other	3 (2.4)
	Not answered	6 (4.8)
Highest level of participation		
	National	44 (35.5)
	International—age group	35 (28.2)
	International—open	37 (29.8)
	Not answered	8 (6.5)
Years competing at Tier 3 or Tier 4 ^e^		
	1 to 3	41 (33.1)
	3 to 5	37 (29.8)
	5 to 10	24 (19.4)
	10 plus	14 (11.3)
	Not answered	8 (6.5)

^a^ School years are described per the New Zealand Qualifications Authority (NZQA); ^b^ includes athletes who specified also needing to purchase their own food for training; ^c^ responses included: dairy-free (including lactose sensitivity and intolerance) (*n* = 8), gluten-free (including wheat-free and gluten intolerance) (*n* = 5), vegetarian (includes pollotarian, pesco–pollo vegetarian) (*n* = 5), diabetes (*n* = 3), coeliac disease (*n* = 2), low iron (*n* = 2), nut allergy (*n* = 2), other food allergies, environmentally conscious, no fowl meat (all *n* = 1); ^d^ heavyweight (*n* = 27), lightweight, coxswain (both *n* = 3); athletics includes running (*n* = 5), track (*n* = 4), jumping, sprinting (all *n* = 1); cycling includes track, road, (both *n* = 4), and mountain bike (*n* = 2), unknown (*n* = 1); skating includes artistic roller skating (*n* = 3), figure skating (*n* = 4), inline hockey (*n* = 1); other includes para-cycling, surf lifesaving, tennis (all *n* = 1); ^e^ tier 3 = highly trained/competing at national level, tier 4 = elite/competing at international level.

**Table 2:** Most preferred people to learn with in NE sessions (*n* = 124) ^a^.

Variable	*n* (%)
Athletes of the same sporting calibre	76 (61.3)
Athletes of similar age	65 (52.4)
Teammates	64 (51.6)
Coaches	64 (51.6)
Any athlete interested in performance nutrition	50 (40.3)
Athletes of the same gender	41 (33.1)
Athletes competing in the same sport	40 (32.3)
Any person interested in performance nutrition	30 (24.2)
Personal support members (e.g., partner, family, parents)	29 (23.4)
Other ^b^	1 (0.8)

^a^ Multiple responses allowed; missing information (*n* = 5, 4.0%); ^b^ other response: ‘Dietitians that know a lot about the specific sport’.

**Table 3:** Session duration and contact frequency (*n* = 124).

Session Variable		In Person Group Session, *n* (%)	Online Session, *n* (%)
People in session ^a^			
	1–5	39 (31.5)	21 (16.9)
	6–10	55 (44.4)	39 (31.5)
	11–20	26 (21.0)	38 (30.6)
	21–30	4 (3.2)	13 (10.5)
	31–40	0 (0.0)	3 (2.4)
	41–50	0 (0.0)	2 (1.6)
	>50	0 (0.0)	7 (5.6)
Session duration			
	<15 min	0 (0.0)	2 (1.6)
	15–30 min	20 (16.1)	38 (30.6)
	31–60 min	79 (63.7)	73 (58.9)
	61–90 min	22 (17.7)	10 (8.1)
	91 min–½ day	2 (1.6)	0 (0.0)
	Full day	1 (0.8)	1 (0.8)
Session frequency			
	<1 every 2 months	15 (12.1)	20 (16.1)
	1 every 2 months	39 (31.5)	33 (26.6)
	1 per month	45 (36.3)	48 (38.7)
	2 per month	19 (15.3)	15 (12.1)
	1 per week	6 (4.8)	8 (6.5)
	2 per week	0 (0.0)	0 (0.0)
Total sessions ^a^			
	1–5	47 (37.9)	44 (35.5)
	6–10	47 (37.9)	47 (37.9)
	11–20	21 (16.9)	24 (19.4)
	21–30	5 (4.0)	6 (4.8)
	31–40	2 (1.6)	1 (0.8)
	41–50	1 (0.8)	1 (0.8)
	>50	1 (0.8)	0 (0.0)

^a^ Missing information for online session preferences: (*n* = 1, 0.8%).

**Table 4:** Preferred qualities in a NE facilitator (*n* = 124) ^a^.

		*n* (%)
Personality traits		
	Credible	91 (73.4)
	Relatable	83 (66.9)
	Likeable	83 (66.9)
	Non-judgmental	82 (66.1)
	Organised	79 (63.7)
	Friendly	71 (57.3)
	Trustworthy	71 (57.3)
	Motivated	66 (53.2)
	Creative	51 (41.1)
	Empathetic	46 (37.1)
	Other ^b^	3 (2.4)
Relatability		
	Knowledge of the sport	106 (85.5)
	Willing to learn more about the sport	88 (71.0)
	Prior experience as an athlete	72 (58.1)
	Previous experience as an athlete in the sport	27 (21.8)
	Athletic physical appearance	16 (12.9)
	Same gender	11 (8.9)
	Similar in age	6 (4.8)
	Does not need to be relatable	3 (2.4)
	Other	1 (0.8)
Credibility		
	Experience in sports nutrition	95 (76.6)
	Registered nutrition professional	84 (67.7)
	Experience in nutrition with athletes of similar calibre	73 (58.9)
	Experience in nutrition with similar sports	57 (46.0)
	Bachelor’s degree in nutrition	56 (45.2)
	Experience in nutrition with the sport	44 (35.5)
	Experience in general nutrition	37 (29.8)
	Does not need to be credible	0 (0.0)
	Other	1 (0.8)

^a^ Multiple responses allowed; ^b^ other responses: “Knowledgeable”, “up to date on new nutrition gains”, and “relevant”.

## Data Availability

The data presented in this study are available on request from the corresponding author. The data are not publicly available due to not being covered in the ethics application and approval.
